# Facile Access to Salt‐Resistant Polyzwitterionic Hydrogel Evaporator via In Situ Frontal Curing‐3D Printing Strategy

**DOI:** 10.1002/advs.202514099

**Published:** 2025-11-16

**Authors:** Ya‐Lan Zhao, Jia‐Le Lu, Yong‐Chun Hou, Liangliang Zhu, Qing Li, Su Chen

**Affiliations:** ^1^ State Key Laboratory of Materials‐Oriented Chemical Engineering, College of Chemical Engineering and Jiangsu Key Laboratory of Fine Chemicals and Functional Polymer Materials Nanjing Tech University Nanjing 210009 P. R. China

**Keywords:** 3D printing, evaporator, frontal polymerization, hydrogel, in situ curing

## Abstract

Freshwater crisis has emerged as a global problem, which has significant negative impact on human production and life. Thus, material design and fabrication are extremely necessary for high salt tolerance and highly efficient evaporation in freshwater production. In this work, polyzwitterionic hydrogel is designed by an in situ frontal curing‐3D printing strategy, which greatly saves times and energy, while ensuring high fidelity and integrity of the printed structure. The polyzwitterionic hydrogel has anti‐polyelectrolyte effect and boosted hydration, showing a higher intermediate water/free water ratio and lower evaporation enthalpy. Meanwhile, the macro‐channels formed by 3D printing and inherent microchannels in hydrogels are synergistically enhancing the water transport performance. As a consequence, the hydrogel exhibits a high evaporation rate of 4.02 kg·m^−2^·h^−1^ in 15 wt.% NaCl solution with excellent salt tolerance. This work provides an in situ frontal curing‐3D printing strategy for the preparation of highly efficient and salt‐tolerant polyzwitterionic hydrogel evaporator, which would guide the development of advanced hydrogel evaporator in a facile fashion.

## Graphical abstract

Polyzwitterionic hydrogel evaporator is developed by a facile in situ curing 3D printing strategy. It shows higher intermediate water/free water ratio in high‐salinity brine, leading to increased amount of activated water and lower evaporation enthalpy, which exhibits high evaporation efficiency and satisfactory salt tolerance.

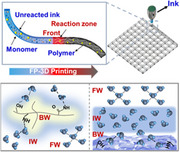

## 1 Introduction

Methods allowing the water resources to be produced in an effective and low‐cost fashion is crucial due to the severe water shortages.^[^ ^1^, ^2^ ^]^ Recently, water desalination has emerged as a green sustainable strategy to ease the water crisis, which converts seawater into fresh water through solar energy.^[^ ^3^ ^]^ Scientists have long endeavored to optimize the evaporation efficiency by developing high‐performance photothermal materials and new evaporator structure,^[^ ^4^, ^5^ ^]^ regulating the water pumping pathway, heat distribution, etc. For instance, a variety of photothermal materials have be used to construct evaporator, including metallic,^[^ ^6^ ^]^ and carbon‐based materials (polydopamine, carbon black and lignin),^[^ ^7^, ^8^ ^]^ etc. Notably, porous hydrogel materials can enhance the reflection and scattering of light, which are increasingly appreciated as a promising solar interface evaporation material.^[^ ^9^, ^10^ ^]^ By regulating the layered nanostructures and microporous structures of hydrogels, the water state can be controlled to reduce the enthalpy of evaporation, thus leading to a higher evaporation rate.^[^ ^11^, ^12^ ^]^ Besides, it has been proved that the 3D structure is promising to break through current theoretical limits to achieve higher evaporation rate, as compared to the normal 2D planes.^[^ ^13^ ^]^ 3D evaporators show distinct advantages of the complete isolation from water, low temperature of side surfaces, enhanced illumination area and reduced diffuse reflection, giving rise to a significant improvement of evaporation performance.^[^ ^14^ ^]^ Zhang et al.^[^ ^15^ ^]^ designed the conical array surface structure to enhance multiple surface reflections and the light absorption capacity, achieving a seawater evaporation rate of 1.96 kg·m^−2^·h^−1^. By mimicking the tree transpiration and hierarchical porous structure, Zhang et al.^[^ ^16^ ^]^ printed bimodal porous structure (high degree interconnectivity wick channels and inherent open microchannels) and achieved an evaporation rate of 2.13 kg·m^−2^·h^−1^. Despite dramatic progresses have been made, the salt crystallization on the evaporator surface becomes a key challenge limiting the long‐term use of solar evaporators. The salt crystals reduce the absorption of sunlight, block the channel of steam evaporation, as well as reduce the effective evaporation area, resulting in a serious decline in the photothermal conversion efficiency and evaporation rate of the evaporator.

Great efforts have been devoted to explore salt‐resistant evaporator to reduce salt accumulation. On one hand, researchers focused on structural design including geometric structure (restricting salt crystals at the edge) and Janus structure (blocking salt crystals underneath the surface).^[^ ^17^, ^18^, ^19^, ^20^ ^]^ For instance, Xu et al.^[^ ^18^ ^]^ designed a Janus solar evaporator, where the hydrophilic bottom layer contributed to continuous water supply, while the top layer was used for light absorption and water evaporation. The salt crystals are confined to the region of the hydrophilic/hydrophobic interface, thus both light absorption and water evaporation rates on the hydrophobic surface remain stable. Liu et al.^[^ ^19^ ^]^ prepared a 3D hydrogel evaporator with an envisaged vertical radiant structure, realizing high‐rate and stable solar desalination even in high‐salinity brine is achieved. Wang et al.^[^ ^20^ ^]^ developed a conical array‐structured solar water evaporator and realized stable salt resistance under the synergism of the anti‐polyelectrolyte effect and Marangoni flow, achieving a water evaporation rate of 2.57 kg·m^−2^·h^−1^ in 10 wt.% NaCl aqueous solution. On the other hand, chemical activation of water has been recognized as a new strategy,^[^ ^21^ ^]^ which was best illustrated by the hydrogel ionization and polyzwitterions.^[^ ^22^, ^23^, ^24^ ^]^ There is a large amount of oppositely ionic groups in the polyzwitterions, showcasing anti‐polyelectrolyte effect.^[^ ^25^ ^]^ In brine, the salt ions screen the electrostatic interactions, dissociates the cross‐links and expand the polymer chains, thereby exposing more hydratable ionic groups and boosting water flux. In this case, the evaporation performance in brine is highly improved.^[^ ^26^, ^27^ ^]^ In spite of these indisputable achievements, the tedious and complex approach seems to be far from easy to implement. Therefore, it is in urgent demand to develop a facile and common strategy toward high‐performance salt‐resistant hydrogel evaporator.

In terms of the construction of 3D evaporators, 3D printing, especially the direct ink writing (DIW) provides great potentials as a versatile, applicable and low‐cost strategy.^[^ ^28^, ^29^ ^]^ DIW has broad applicability without material limit, which is applicable to shear‐thinning fluid. However, it also remains vital challenges, such as suffering from collapse due to the gravity and the requirement of post‐processing, which is difficult to ensure the fidelity and integrity of the printed structures.^[^ ^30^ ^]^ Meanwhile, it is usually accompanied with time‐ and energy‐consumption. To overcome these drawbacks, an in situ curing method during 3D printing process is highly desirable. Frontal polymerization (FP), as a self‐sustained reaction, provides new opportunities for 3D printing.^[^ ^31^, ^32^ ^]^

FP allows the in situ curing of monomers through the propagating of a localized reaction zone, where the reaction heat drives further polymerization rather than requiring external energy input. The concept of in situ curing 3D printing has been demonstrated through frontal ring‐opening metathesis polymerization (FROMP) of dicyclopentadiene for thermosetting polymers.^[^ ^33^, ^34^ ^]^ Compared with conventional bulk thermal polymerization, FP offers several distinct advantages. (1) Low curing energy consumption: For instance, the energy requirements of the FROMP were reduced by more than ten orders of magnitude compared with traditional bulk thermal curing. (2) High curing efficiency: Conventional bulk thermal polymerization typically requires 5–20 h for curing, whereas FP can complete curing within just 10–30 min.^[^ ^35^ ^]^ (3) Good product uniformity: the FP process is relatively smooth and moderate, thereby leading to good product uniformity, e.g. uniform porous structure of hydrogel.^[^ ^36^, ^37^ ^]^ Besides, epoxy resin‐based thermosets and epoxy‐vinyl ether structures were also developed using FP‐3D‐printing method.^[^ ^38^ ^]^ In our previous work, we have expanded this facile strategy for hydrogel system.^[^ ^39^ ^]^ It offers a simple and energy‐saving in situ curing 3D printing pathway, not only ensuring the fidelity and integrity of hydrogel evaporator, but also shedding new light on the development of DIW technique. Notably, this FP‐3D‐printing method toward soft hydrogels is still in its early stage. Continuous research works are necessary to boost the prosperous development of this method.

**Scheme 1:**
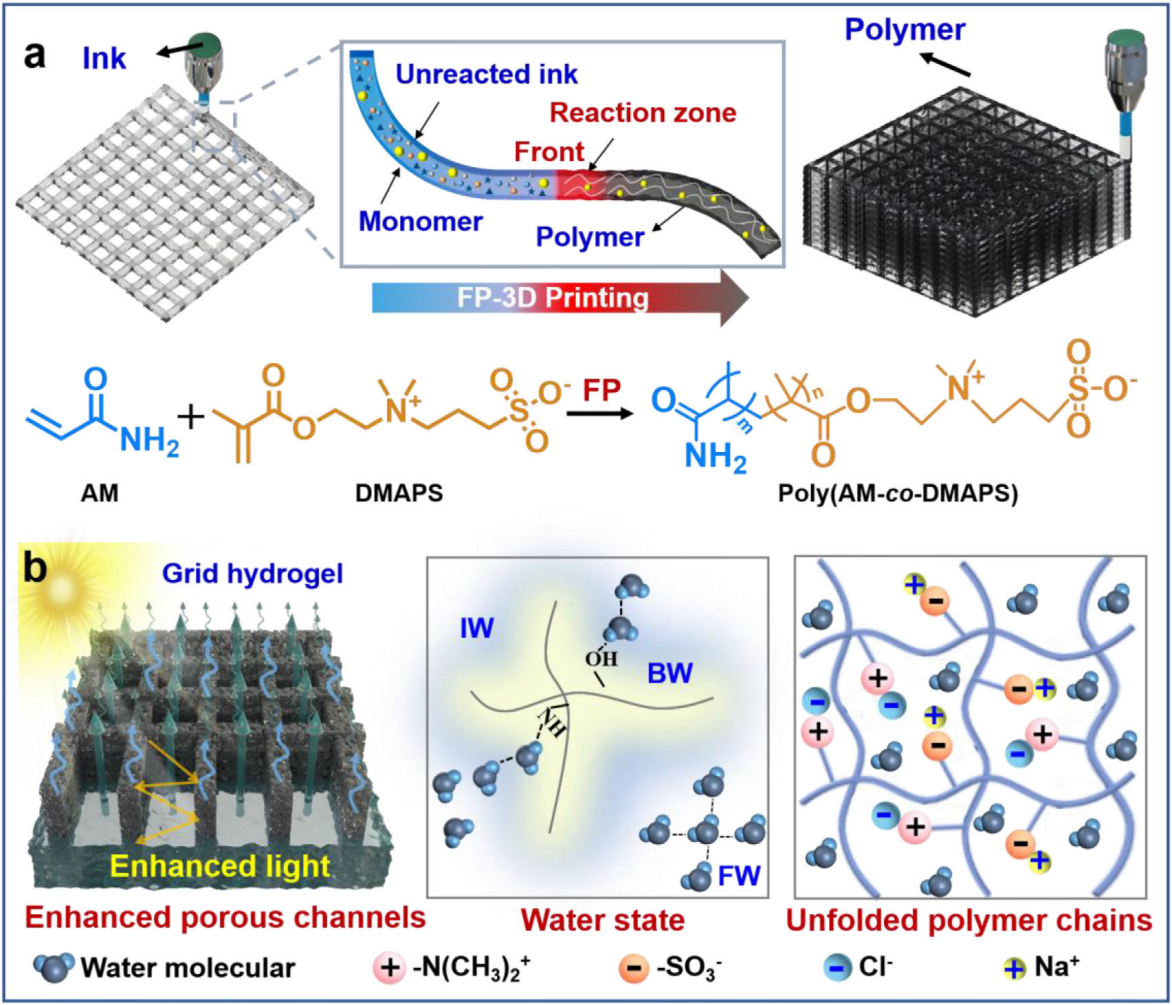
a) Schematic illustration of the in situ curing FP–3D printing strategy of the printable and polymerizable ink toward grid hydrogel evaporator. b) Evaporation mechanism of the salt‐resistant grid hydrogel in NaCl solution.

In this work, we develop a FP–3D printing strategy toward salt‐resistant 3D hydrogel evaporators, which shows a high evaporation rate of 4.02 kg·m^−2^·h^−1^ in high‐salinity brine. This work shares the following advantages. (1) We design a printable and curable ink, where the zwitterionic monomer ([2‐(Methacryloyloxy)ethyl]dimethyl‐(3‐sulfopropyl) = DMAPS) and highly reactive monomer (acrylamide = AM) were selected. The in situ curing process, namely FP–3D printing, is successfully realized for the fabrication of 3D hydrogel patterns. Following the 3D printing trajectory, this real‐time curing occurs without external energy input. This feature brings great convenience to the superiority of high fidelity and integrity of the printed structure, and meanwhile it is time and energy saving compared with the current extrusion printing technique (**Scheme** **1**a). (2) In virtue of the anti‐polyelectrolyte effect of the polyzwitterionic hydrogel, the hydrogel evaporator shows enhanced swelling behavior and boosted hydration in brine, which has higher intermediate water (IW)/free water (FW) ratio with higher DMAPS content. Particularly, the IW/FW ratio in even higher in high‐salinity brine (up to 1.36), leading to the increased amount of activated water and lower evaporation enthalpy (1453.8 J g^−1^). (3) The printed hydrogel evaporator features macro‐channels formed by 3D printing and inherent microchannels in hydrogels, which could enhance the water transport performance. Based on the anti‐polyelectrolyte effect, porous structure and boosted hydration (Scheme 1b), the 3D hydrogel evaporator shows a high evaporation rate of 4.02 kg·m^−2^·h^−1^ in 15 wt.% NaCl solution. In addition, it shows good salt tolerance, where the Na^+^ and Cl^−^ are attracted through electrostatic interaction,^[^ ^40^, ^41^ ^]^ avoiding the crystallization on the evaporator surface. This work provides an in situ curing FP–3D printing method for the fabrication of salt‐resistant polyzwitterionic hydrogel, which will stimulate the development of DIW toward high‐performance 3D hydrogel evaporators.

## 2 Results and Discussion

### 2.1 FP‐3D Printing Toward Hydrogel Evaporator

**Figure 1:**
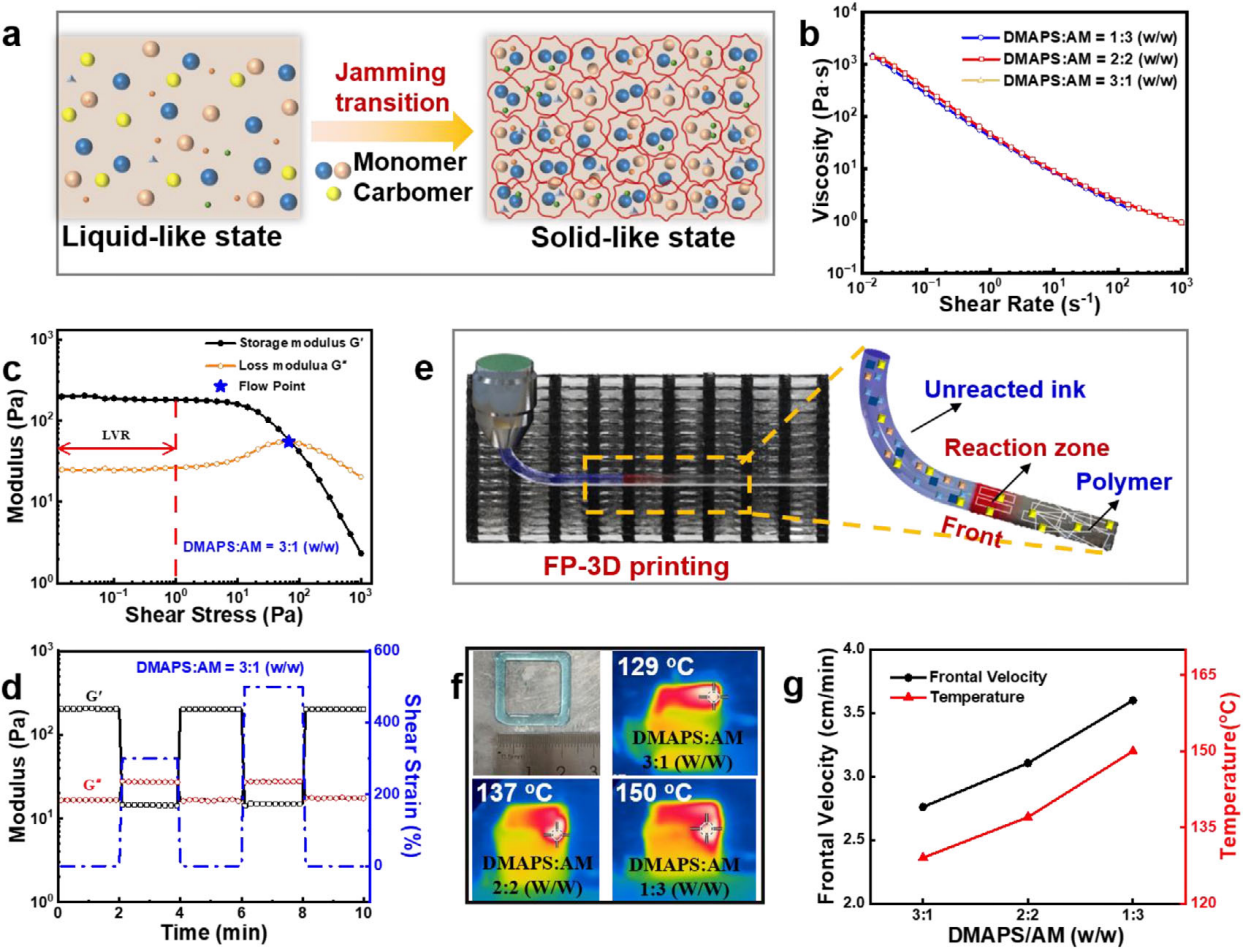
a) Schematic illustration of the jamming transition of the ink. b) Relationship between viscosity and shear rate of ink with different DMAPS/AM mass ratios of 1:3, 2:2 and 3:1 (w/w). c) Storage modulus (G') and loss modulus (G“”) of ink versus shear stress at DMAPS/AM mass ratio of 3:1 (w/w). d) Storage modulus G' and loss modulus G“” under dynamic cyclic strain sweeps at DMAPS/AM mass ratio of 3:1 (w/w). e) Schematic illustration of in situ FP‐3D printing process. f) IR thermal images during the FP‐3D printing process at different DMAPS/AM mass ratios. g) Frontal velocity and *T_max_* during FP‐3D printing process at DMAPS/AM mass ratios of 1:3, 2:2 and 3:1 (w/w).

For the purpose of developing a real‐time curing FP‐3D printing strategy toward salt‐resistant 3D hydrogel evaporator, we first design the printable and polymerizable ink, where DMAPS and AM are chosen as reactive monomers, while Carbomer 940 and prussian blue (PB) serve as thickening agent and photothermal material, respectively. Specially, Carbomer 940 could be swollen in solution to form stacked microgels, endowing the ink with excellent injectability and printability (**Figure** **1**a). AM acts as highly reactive monomer to provide abundant of reaction heat, which is beneficial for sustaining the self‐propagating FP process. DMAPS, as a zwitterionic co‐monomer, which has cationic and anionic functional groups that can form electrostatic interaction with the positive and negative ions in salt solution, rendering the hydrogel with unique anti‐polyelectrolyte effect toward high‐performance salt‐resistant evaporators.^[^ ^24^, ^40^, ^41^ ^]^ As a photothermal absorber, PB has an extremely wide absorption spectrum from visible light to near‐infrared. It has extremely high photothermal conversion efficiency and excellent photothermal stability, as well as superiority of non‐toxicity and environmentally friendless, which shows potentials to construct evaporator.^[^ ^42^ ^]^ The rheological properties of ink play a crucial role in the printing process. As shown in Figure 1b, all the inks with different DMAPS/AM mass ratios have high viscosity at low shear rates, which can maintain a stable structure. As the shear rate gradually increases from 0.01 to 1000 s^−1^, all the viscosity curves of three inks show the same decline trend with about 3 orders of magnitude. The unique shear‐thinning behavior enables smooth and continuous extrusion of ink from the print nozzle while remaining stable after extrusion. Figures 1c and S1 (Supporting Information) shows the storage modulus (G') and loss modulus (G″) variation of the ink with shear strain. The inks exhibit a linear viscoelastic response in the range of low shear stress, where G' is nearly an order of magnitude higher than G″. In this case, the ink exhibits a “solid‐like” behavior, which can maintain structural stability after extrusion. After the linear viscoelastic region (LVE), the ink yields and G' decreases continuously, indicating that the internal structure of the ink is gradually destroyed. When G″ is larger than G', the ink appears to flow. If the ink is still flowing when it is extruded on the printing platform, the printing process can't continue. As thus, we conducted the dynamic cyclic stress sweep tests to explore the structural recovery ability of the ink after extrusion. As shown in Figures 1d and S2 (Supporting Information), the ink undergoes a cyclic test from low strain to high strain. Interestingly, the inks are able to quickly recover the G' at the end of each high strain, exhibiting a high structural recovery rate of 96%. The rapid recovery of storage modulus G' enables the excellent printability of the ink, meanwhile it guarantees a high fidelity of the printed patterns.

We then focused on the exploration of the FP‐3D printing process. The pure FP process was proved in glass tube (diameter of 10 mm), as shown in Figure S3 (Supporting Information). All the frontal position‐time curves are well fitted in straight lines, indicating a constant frontal velocity. When the DMAPS/AM mass ratios ranging from 3:1 to 1:3 (w/w), the frontal velocity increases from 1.11 to 1.58 cm min^−1^, while the frontal temperature (*T_max_*) raises from 127 to 146 °C, respectively. In terms of the FP‐3D printing process, the end of the printed pattern is heated once the ink is extruded, until a front is formed. Then the front could travel along the printing trajectory, converting monomers into polymers by using the heat release of the polymerization, rather than external energy supply. Figure 1e clearly illustrates the FP‐3D printing process, where a propagating front is following close behind the printing trajectory, leaving behind polymer and unreacted monomer in its path. The FP‐3D printing process is best illustrated by the thermal IR images in Figure 1f. A white area indicates a maximum temperature, that is the reaction zone of the propagating front. Behand the front, the temperature declines due to thermal diffusion. The *T_max_* confirms the occurrence of pure FP during the 3D printing process. Figure 1g displays the frontal velocity and *T_max_* during FP‐3D printing process. It is found that both frontal velocity and *T_max_* are proportional to AM content, which could be attributed to the higher polymerization enthalpy of AM.^[^ ^39^ ^]^

### 2.2 Anti‐Polyelectrolyte Effect and Boosted Hydration Behavior of Hydrogel Evaporator

**Figure 2:**
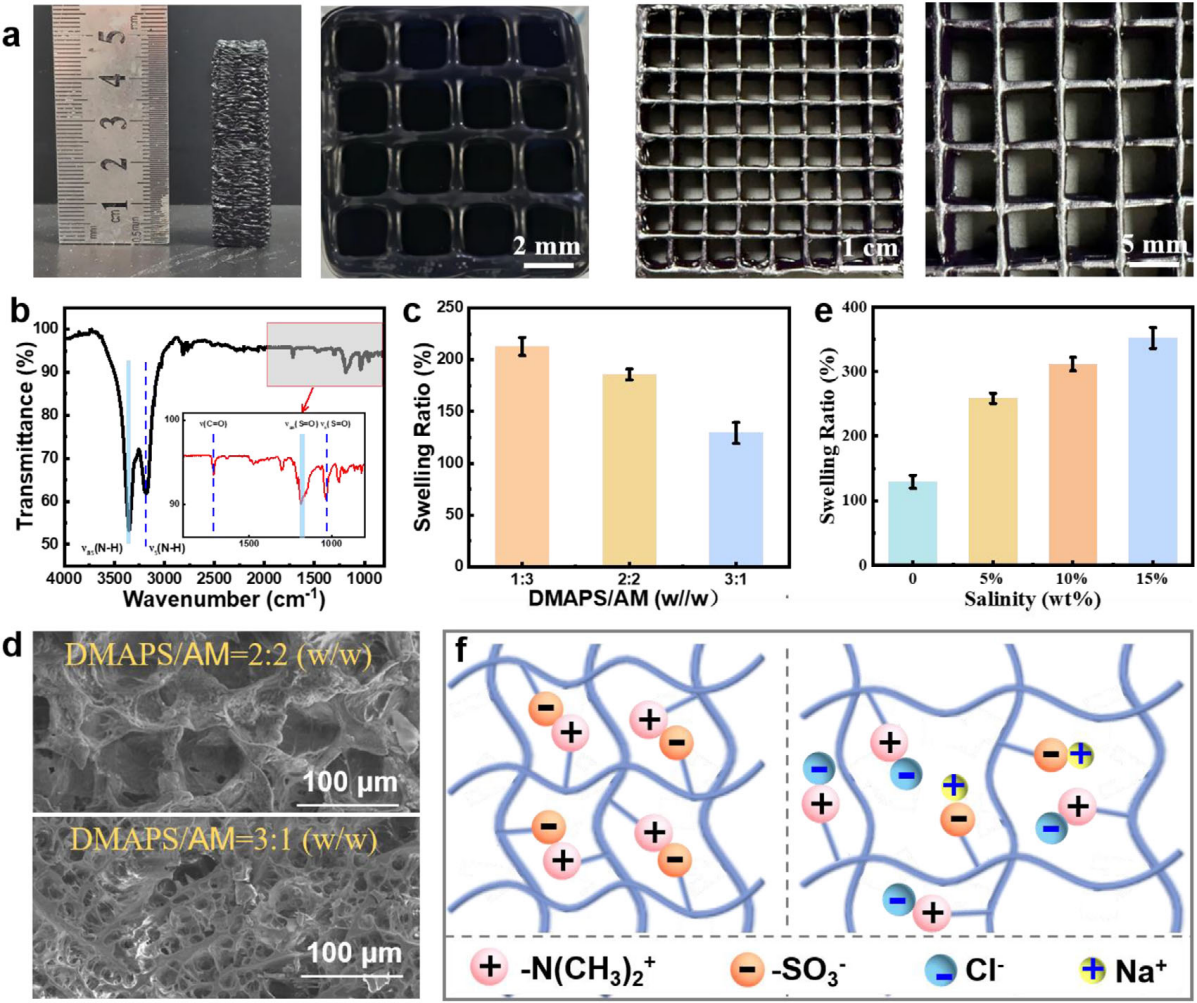
a) Photographs of the hydrogel samples prepared by the FP‐3D printing process. b) FTIR spectrum of poly(DMAPS‐*co*‐AM)/PB hydrogel. c) ESR of hydrogel at varying DMAPS/AM mass ratios of 1:3, 2:2 and 3:1 (w/w). d) SEM images of hydrogel. e) ESR of hydrogel at varying salinity (DMAPS/AM mass ratios of 3:1 (w/w)). f) Schematic illustration of the polyelectrolyte effect of polyzwitterionic hydrogel.

The FP‐3D printing method is versatile and flexible to adjust the macroporous channel by controlling the printing path. Meanwhile, the printed structure has high fidelity owing to the in situ‐curing process, which efficiently avoids the most common collapse and deformation of printed soft material (**Figure** **2**a). The chemical structure of the as‐printed poly(DMAPS‐*co*‐AM)/PB hydrogel was analyzed by the FTIR spectra, as shown in Figure 2b. Characteristic absorption peaks at 3352.67 and 3181.01 cm^−1^ correspond to the antisymmetric and symmetric stretching vibration of N─H in AM.^[^ ^43^ ^]^ While the peak at 1715 cm^−1^ is assigned to the C═O stretching vibration of carbonyl group. The characteristic peaks at 1188 and 1037 cm^−1^ reflect on the S═O antisymmetric and symmetric stretching vibrations in DMAPS. These characteristic peaks confirm the successful synthesis of the poly(DMAPS‐*co*‐AM)/PB hydrogel. Figures 2c and S4 (Supporting Information) demonstrate the swelling behavior of hydrogels with varying DMAPS/AM mass ratios in water. With increasing DMAPS content, the equilibrium swelling ratio (ESR) shows a declined trend. Typically, the ESR is 212, 185 and 129, corresponding to DMAPS/AM mass ratio of 1:3, 2:2 and 3:1 (w/w), respectively. From the SEM images, it is found that the hydrogel exhibits typical microporous structure with relatively uniform size (Figures 2d and S5, Supporting Information). The pore size decreases with DMAPS content, which is consistent with the swelling behavior. Notably, the FP‐3D printed hydrogel has both macroporous (2 mm) and microporous (20‐100 µm) structures, which would form rich interpenetrating channels to accelerate steam escape and facilitate water transport.^[^ ^44^ ^]^ The water‐transfer capacity of bulk and 3D grid hydrogel was compared by immersing in red ink solutions. It is found that the 3D grid hydrogel exhibits faster water‐transfer capacity, where the red ink can be transferred to the upper surface of the 3D grid hydrogel within ten min (Figure S6, Supporting Information). The water usually requires many h to reach the upper surface of a bulk hydrogel evaporator.^[^ ^45^ ^]^ Notably, the 3D grid hydrogel shows super water‐transfer capacity as compared to bulk hydrogel. Whereas, it is still inferior to nanofiber membrane‐ or aerogel‐based evaporator, where the water transfer could be completed within few seconds or min.^[^ ^46^, ^47^ ^]^ Moreover, we investigated the swelling behavior of the hydrogel (DMAPS/AM mass ratio of 3:1 (w/w)) in brine with different salinity (Figures 2e and S7, Supporting Information). Interestingly, the hydrogel shows enhanced swelling behaviors in brine, generating the largest ESR of 351 in 15 wt.% brine, which is 2.72 times of that in water. The enhanced water‐absorption capacity is ascribed to the anti‐polyelectrolyte effect of the DMAPS, which has a large amount of oppositely charged ionic groups. In brine, electrostatic interaction generates between anionic/cationic salt ions and oppositely charged ionic groups in DMAPS, greatly screening the inter/intra‐chain associates, thereby leading to the expansion of the zwitterionic chains^[^ ^48^, ^49^ ^]^ (Figure 2f). Besides, the water contact angle of the hydrogel decreases with increase of DMAPS content and salinity (Figure S8, Supporting Information), further revealing the salt‐triggered water‐absorption improvement. These results illustrate that the distinct anti‐polyelectrolyte effect favors swelling behavior in high salinity brines, which is beneficial for evaporation performance and salt‐resistance. In addition, the dimensional stability during prolonged immersion was further investigated, as shown in Figure S9 (Supporting Information). It is found that the structural fidelity kept unchanged within 24 h immersion in saline solution, providing potential practicality as water evaporator.

**Figure 3:**
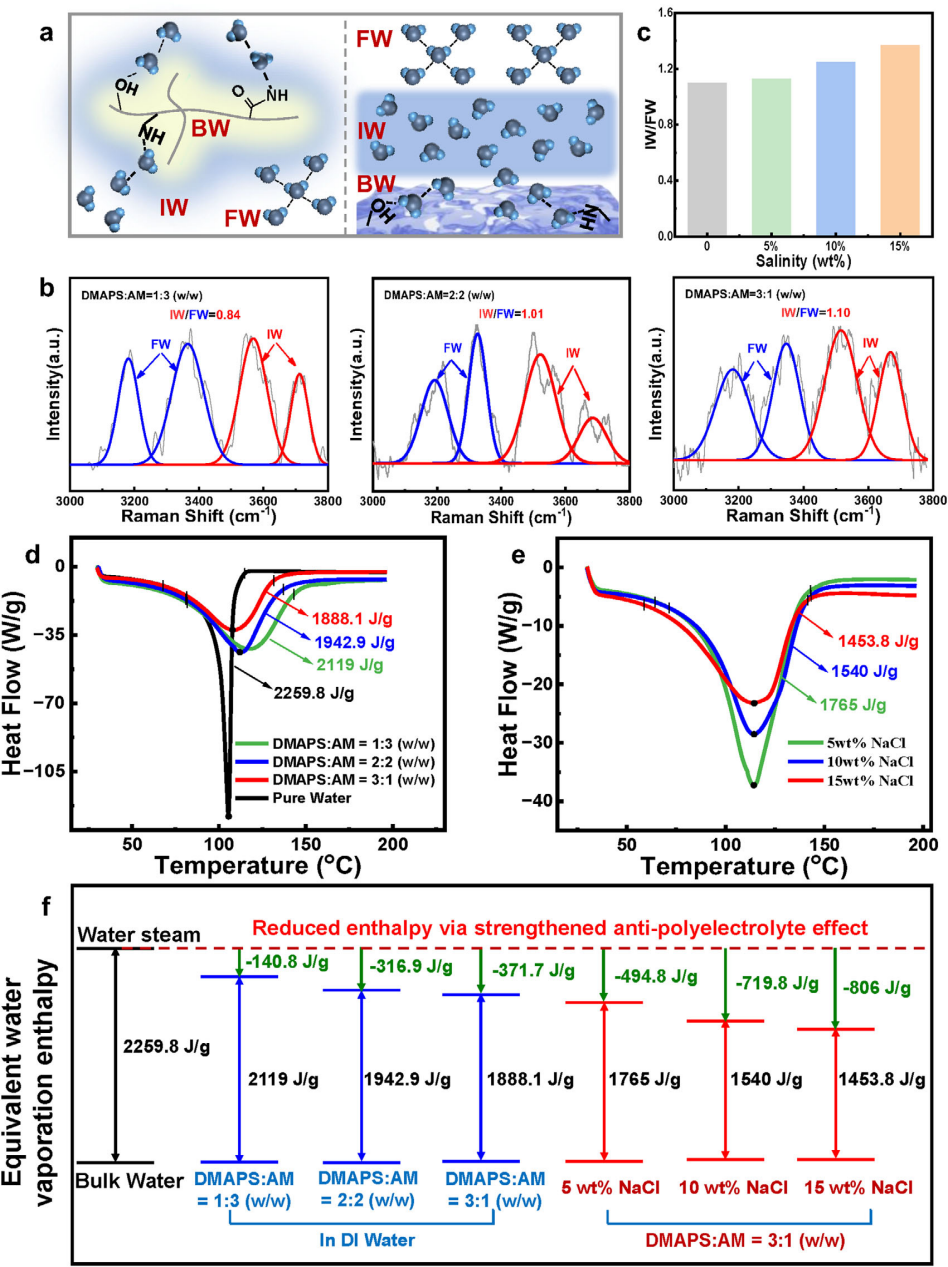
a) Water state diagram inside the poly(DMAPS‐*co*‐AM)/PB hydrogel evaporator. b) Raman spectra of hydrogel at varying DMAPS/AM mass ratios of 1:3, 2:2, and 3:1 (w/w). c) IW/FW ratio of hydrogel at varying salinity (DMAPS/AM mass ratio of 3:1 (w/w)). DSC curve of hydrogel d) at varying DMAPS/AM mass ratios and e) at varying salinity. f) Changing tendency of the evaporation enthalpy of the water in hydrogel at varying DMAPS/AM mass ratios and salinity.

According to the intensity of water‐chains interaction, water contained in the hydrogel is divided into FW, IW and bound water (BW). Among them, IW is identified as activated water, which requires less energy to evaporate, while FW is beneficial for water transport (**Figure** **3**a).^[^ ^24^ ^]^ The water states in the hydrogel was analyzed the by Raman spectra (Figures 3b,c; S10, Supporting Information). Figure 3b shows the Raman spectra of hydrogels with varying DMAPS/AM mass ratios. The peaks at 3182 and 3347 cm^−1^ belong to the FW bonded with four hydrogen bonds, while peaks at 3516 and 3668 cm^−1^ are corresponding to the IW that weakly bonded with adjacent water. It is found that the IW/FW ratio is improved by increasing DMAPS content, which is 0.84, 1.01 and 1.10, corresponding to DMAPS/AM mass ratio of 1:3, 2:2, and 3:1 (w/w), respectively. DMAPS contains strong hydrophilic groups (‐SO_3_^−^, ‐N^+^(CH_3_)_2_), leading to enhanced electrostatic interactions and hydrogen bonds. Thus, water molecules are easily dissociated from the bound water, thereby promoting the evaporation of water.^[^ ^50^, ^51^ ^]^ More interestingly, the water state could be adjusted by zwitterion‐salt‐water interactions. The IW/FW ratio is further enhanced in brine (Figure 3c), reaching to 1.36 in 15 wt.% brine, which indicates that the polyzwitterionic hydrogel is more likely to adsorb ions and interact with water molecules through long‐range electrostatic interactions.^[^ ^41^ ^]^ High IW/FW ratio leads to increased amount of activated water, which suppresses the energy barrier for evaporation.^[^ ^52^ ^]^ The evaporation enthalpy of the water in the hydrogel was further evaluated by DSC measurement, as shown in Figure 3d–f. The results indicate that the evaporation enthalpy of the water in hydrogel is much lower than that of bulk water (2259.8 J·g^−1^). With DMAPS content increasing, the evaporation enthalpy of the water in hydrogel decreases from 2119 to 1888.1 J·g^−1^. Moreover, it significantly drops to an ultra‐low value in 15 wt.% brine (1453.8 J·g^−1^). The results reveal that the zwitterionic monomer could effectively regulate the IW/FW ratio, realizing increased amount of activated water and decreased evaporation energy barrier due to the zwitterion‐salt‐water interactions.

### 2.3 Evaporation Performance of the Grid Hydrogel Evaporator

**Figure 4:**
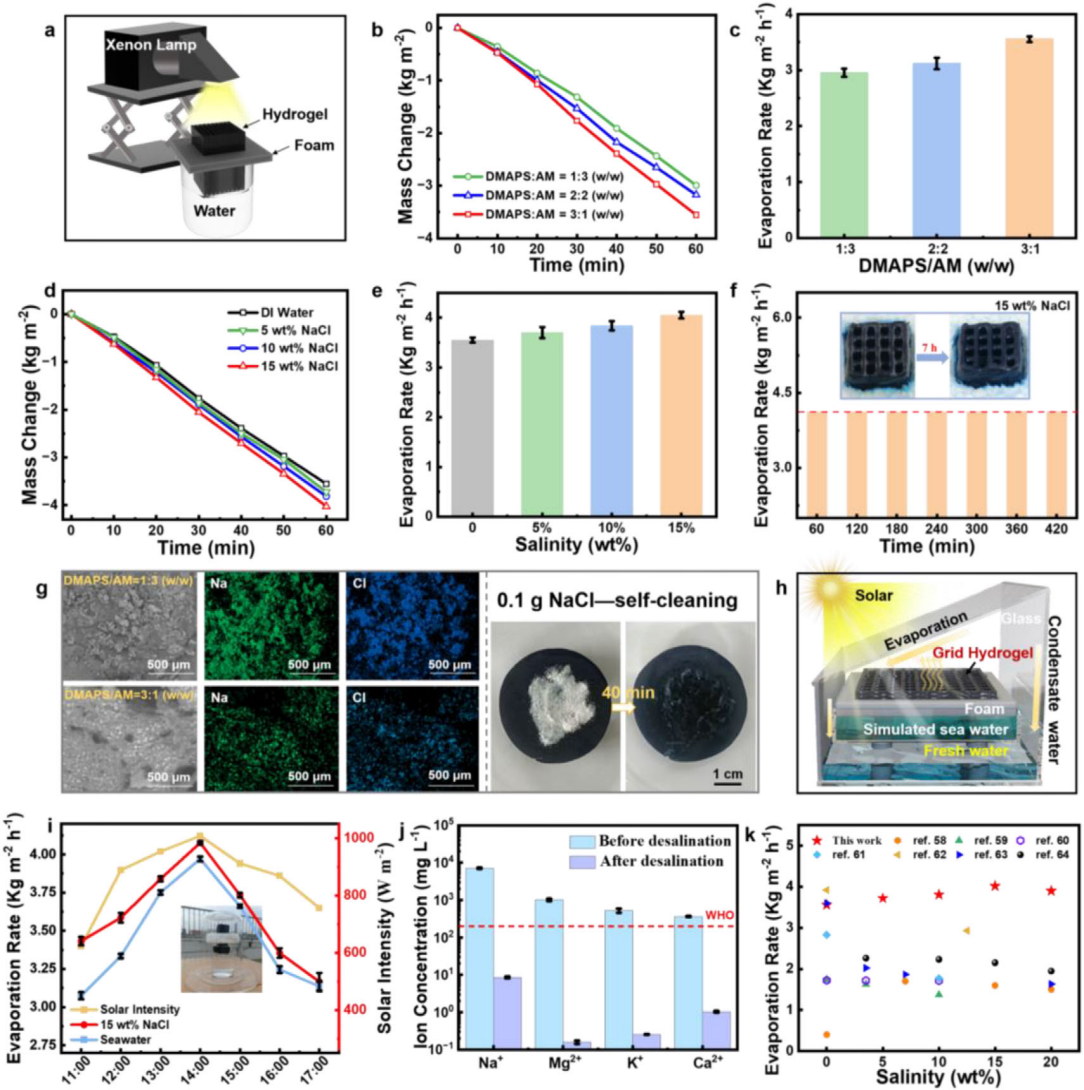
a) Schematic illustration of the water evaporation process. b) Mass changes and c) evaporation rate of hydrogels with different DMAPS/AM mass ratios of 1:3, 2:2, and 3:1 (w/w) in water. d) Mass changes and e) evaporation rate of hydrogels (DMAPS/AM mass ratios of 3:1 (w/w)) at varying salinity. f) Water evaporation rate under continuous 7 h evaporation in 15 wt.% NaCl solution. Inset: digital photographs showing no salt deposition on the hydrogel evaporator surface. g) Left: SEM images and EDS mapping of the hydrogels after evaporation in 15 wt.% brine. Right: Salt self‐cleaning behavior of the hydrogel (DMAPS/AM mass ratios of 3:1 (w/w)). h) Schematic illustration of outdoor evaporation device for simulated seawater and brine evaporation. i) Intensity of solar irradiance and outdoor seawater evaporation performance of the hydrogel under natural sunlight (DMAPS/AM mass ratios of 3:1 (w/w)). Inset: digital photograph of an outdoor evaporation device. j) Ca^2+^, K^+^, Mg^2+^ and Na^+^ concentration of the seawater before and after desalination. k) Comparison of evaporation rate of the hydrogel in different concentrations of NaCl solution with relevant literatures.

The evaporation performance of the grid hydrogel evaporator was thoroughly investigated in pure water and salt water. PB as a photothermal material, which harvests natural sunlight and converts it to thermal energy. The light absorption ability was investigated by UV‐vis‐NIR spectra. An excellent light absorption capacity of 91.84% is achieved in the full solar spectral range of 250–2500 nm (Figure S11a, Supporting Information), which provides advantages for water evaporation. In addition, it is found that the photothermal performance of samples prepared under high temperature (FP method) and low temperature (spontaneous polymerization) remained almost unchanged, indicating the excellent photothermal stability of PB (Figure S11b, Supporting Information). By simulating sunlight exposure at a light intensity of 1 kW m^−2^ under xenon lamps indoors (**Figure** **4**a), we recorded the surface temperature and weight loss over time. It is found that the surface temperature rises sharply within the first 5 min due to the excellent photothermal conversion ability of PB (Figure S12, Supporting Information). In addition, the weight loss shows linear relationship with time, indicating that the evaporation rate is stable. When the DMAPS/AM mass ratio is 3:1 (w/w), the weight loss reach a maximum value with the optimal water evaporation rate of 3.55 kg·m^−2^·h^−1^ (Figure 4b,c). We further investigated the evaporation performance of the hydrogel (DMAPS/AM mass ratio is 3:1 (w/w)) in salt solution at 1 kW m^−2^ light intensity. It should be noted that the evaporation rate in salt solution (NaCl solution with concentration of 5, 10, and 15 wt.%) is higher than that in pure water. A maximum evaporation rate of 4.02 kg·m^−2^·h^−1^ is achieved in 15 wt.% NaCl solution (Figure 4d,e). More importantly, in the continuous evaporation process for 7 h, the evaporation rate is maintained at an average of 4.02 kg·m^−2^·h^−1^. And there is no salt deposition on the evaporator surface, indicating outstanding salt‐resistant performance (Figures 4f and S13, Supporting Information). Besides, SEM images and EDS mapping of the hydrogels after evaporation in 15 wt.% brine give more conclusive evidence (Figure 4g, left). It is found a small amount of salt ions inside the hydrogel (DMAPS/AM mass ratio of 3:1 (w/w)), which is superior to that of the sample (DMAPS/AM mass ratio of 1:3 (w/w)), appearing agglomerated salt crystals. In order to further verify the salt‐resistance, a small amount of NaCl crystal (0.1 g) was placed on the surface of the hydrogel. Interestingly, the NaCl crystal can be dissolved and diffused into the bulk water within 40 min due to the rapid capillary wicking effect of the porous hydrogel (Figure 4g, right), showing a certain salt rejection performance. Due to the anti‐polyelectrolyte effect of the hydrogel, the polymer chain becomes more stretchable in saltwater.^[^ ^24^ ^]^ In this case, the Na^+^ and Cl^−^ ions in the salt solution are attracted on the functional groups with opposite charges, and thus salt crystallization is impeded on the surface of the evaporator.

Outdoor seawater evaporation performance plays an important role for the practical application of hydrogel evaporator. Therefore, we designed an outdoor evaporation device to test real‐time light intensity and simulated seawater evaporation rate on a sunny day in June 2025, Nanjing, China (Figure 4h). The outdoor environmental parameters such as temperature, humidity and wind speed were recorded in Figure S14 (Supporting Information). As illustrated in Figure 4i, the intensity of solar irradiance increased first and then decreased with time from 11:00 a.m. to 17:00 p.m, reaching a maximum value at 14:00 p.m. It is found that the evaporation rate of hydrogel evaporator showed a similar tendency, which was up to 3.97 kg·m^−2^·h^−1^ in seawater and 4.07 kg·m^−2^·h^−1^ in 15 wt.% brine at 14:00 p.m. Besides, we investigated the evaporation performance of the hydrogel toward seawater. After evaporation, the concentration of Na^+^, K^+^, Mg^2+,^ and Ca^2+^ is 7.6483, 0.5273, 0.3458, and 0.4729 mg L^−1^, respectively, which is lower than the WHO standard value for freshwater (Figure 4j).^[^ ^53^ ^]^ It is worth noting that the evaporation rate at 14:00 in outdoor experiments is slightly larger than that under indoor condition. In the indoor environment, the energy source is only simulated sunlight (≈1 kW·m^−2^), which is a controlled single energy input. While in the outdoor environment, the real full‐spectrum sunlight is changed with time, where the solar intensity (1008.3 kW·m^−2^, 14:00) could be larger than one sun (Figure 4a). Besides, the hydrogel evaporator also absorbs long‐wave infrared radiation emitted from surrounding warm objects, which further improves the evaporation capacity. Moreover, in most practical cases, evaporators may encounter complex wastewater, such as organic dye wastewater. Thus, we simulated industrial dye wastewater using Rhodamine B, methyl orange, and methylene blue aqueous solutions to evaluate the wastewater treatment performance of hydrogel evaporators. As shown in the UV‐visible spectrum in Figure S15 (Supporting Information), obvious characteristic peaks are observed before evaporation. Interestingly, the condensed water becomes transparent and the characteristic peaks of dye molecules disappear. The hydrogel possesses cationic and anion groups on the polymer chain, which allows the formation of strong electrostatic interactions between the sulfonic acid groups and the tertiary amine groups, contributing to efficient dye wastewater purification. All these results give strong evidence that the grid hydrogel evaporator holds great potential both in solar desalination and organic dyes wastewater purification, which could sustain long‐term usage in salt solution. Moreover, we compared the evaporation performance of the as‐prepared grid hydrogel with other 3D structures in literatures.^[^ ^54^, ^55^, ^56^, ^57^, ^58^, ^59^, ^60^ ^]^ By virtue of the anti‐polyelectrolyte effect, the grid hydrogel shows high IW/FW ratio of 1.36 and low evaporation enthalpy of 1453.8 J·g^−1^ in 15 wt.% brine, leading to comparable or even higher evaporation rate in high‐salinity brine (Figure 4k).

## 3 Conclusion

In summary, we develop a facile in situ curing 3D printing strategy to prepare polyzwitterionic hydrogel evaporator, which simultaneously achieves high evaporation efficiency and satisfactory salt tolerance. This in situ curing 3D printing strategy combines FP with 3D printing, showing distinct advantages of time‐ and energy‐saving, as well as superiority of high fidelity and integrity of the printed structure. Zwitterionic monomer DMAPS could effectively regulate the water status in the hydrogel, leading to boosted hydration, higher IW/FW ratio due to electrostatic interactions and hydrogen bonds. More interestingly, the IW/FW ratio is even higher in high‐salinity brine (IW/FW ratio of 1.36 in 15 wt.% brine), leading to the increased amount of activated water and lower evaporation enthalpy (1453.8 J·g^−1^). In this case, a high evaporation rate of 4.02 kg·m^−2^·h^−1^ is achieved in 15 wt.% brine. There is no salt accumulation in continuous evaporation of 7 h, indicating that the polyzwitterionic hydrogel possesses salt‐impeding capability. Zwitterionic DMAPS endows the hydrogel evaporator with anti‐polyelectrolyte effect, where the electrostatic interaction between anionic/cationic groups and salt ions is beneficial to the swelling ability in high‐salinity brine and avoid salt crystallization on the evaporator surface. In the outdoor experiment, a maximum evaporation rate of 3.97 and 4.07 kg·m^−2^·h^−1^ is achieved in simulated seawater and 15 wt.% brine, respectively. This work provides a facile in situ curing FP‐3D printing method toward rapid synthesis of polyzwitterionic hydrogel evaporator with high evaporation efficiency and satisfactory salt tolerance, which is meaningful to promote hydrogel evaporator design and fabrication in a facile pathway.

## Conflict of Interest

The authors declare no conflict of interest.

## Supporting information



Supporting Information

## Data Availability

The data that support the findings of this study are available from the corresponding author upon reasonable request.
