# Supplementary material for: Facile Access to Salt‐Resistant Polyzwitterionic Hydrogel Evaporator via In Situ Frontal Curing‐3D Printing Strategy
Source: Adv Sci (Weinh). 2025 Nov 16;13(6):e14099. doi: 10.1002/advs.202514099 (PMC12866803; doi:10.1002/advs.202514099)
Supplement: Supplementary file 1 — Supporting Information [file ADVS-13-e14099-s001.docx]

**Supporting Information**

**1. Experimental Section**

***Materials:*** Dimethyl sulfoxide (DMSO), ethylene glycol (EG), [2-(Methacryloyloxy)ethyl]dimethyl-(3-sulfopropyl) (DMAPS), N, N’-methylenebisacrylamide (MBAA), 2,2,6,6-tetramethylpiperidine 1-oxyl free radical (TEMPO), prussian blue (PB), rhodamine B (Rh B), methylene blue (MB), methyl orange (MO) were purchased from Aldrich and used as received. Carbomer 940 and Acrylamide (AM) were purchased from Macklin and used as received. Ammonium persulfate (APS) were purchased from Sinopharm Chemical Reagent Co., Ltd. High-purity water with the resistivity of greater than 18 MΩ·cm was used in the experiments.

***Preparation of 3D printing ink:*** 0.06 g of PB was dissolved in the mixture of DMSO (2 g) and EG (4 g), followed by ultrasonic dispersion for 30 minutes. Then 1 g of AM, 3 g of DMAPS and 0.03 g of MBAA were added in the mixture, forming homogenous solution under magnetic stirring. Afterwards, 0.25 g of Carbomer 940 was added, followed by vigorous stirring at 45 ^o^C to form a transparent solution. Finally, 0.1 g of APS and 0.017 g of TEMPO were added to the cooled mixture (room temperature) to obtain the 3D printing ink. By changing the DMAPS/AM mass ratios, three inks were achieved, where the DMAPS/AM mass ratio is 1:3, 2:2 and 3:1 (w/w), respectively.

***Fabrication of 3D structures by FP-3D printing:*** A microfluidic 3D printer equipped with extrusion system (Nanjing Bell Time Technology Co., LTD) was used. The ink was transferred to a 10 mL syringe, which was fixed on an injection pump. Then the ink was printed at room temperature (the 3D modeling was established by Solidworks 2020 and saved as G-code file). At the beginning of the 3D printing process, the printed pattern was heated by a soldering iron for 20 s. Then a front occurred and the soldering iron was removed. The front travelled along the printing trajectory, converting the monomers into polymers in a real-time curing way.

***Front velocity and temperature tests******: The*** frontal position and corresponding time was recorded and the front velocity was calculated by the slope of the frontal position-time curve. The temperature was recorded by a FLIR E8 IR thermal imager in a fixed point.

***Rheological measurement:*** The rheological properties of the ink were tested by a rheometer (Anton Paar MCR-302) with a plate of 25 mm. Specific test conditions are as follows. (1) Steady-state shear test: shear rate ranging from 0.01 to 1000 s^-1^, temperature of 25 ^o^C. (2) Dynamic oscillation stress sweep tests: shear strain ranging from 0.01% to 1000 %, temperature of 25 ^o^C, frequency of 1 Hz. (3) Dynamic cyclic stress sweep tests: dynamic shear strain ranging from 300 % and 500 %, time of 100 s, temperature of 25 ^o^C, frequency of 1 Hz.

***Differential scanning calorimetry (DSC) measurement:*** The evaporation enthalpy of hydrogel and bulk water was measured by DSC (TA Q2000). The test was conducted from 30 to 200 ^o^C with heating rate of 10 ^o^C /min and nitrogen flow rate of 50 mL/min.

***Raman spectra characterization:*** The type of water molecule was analyzed through Raman microscope (HORIBA, LABRAM HR800) at 514 nm.

***Scanning electron microscopy (SEM) measurement:*** The morphologies of the hydrogel samples were observed by the scanning electron microscopy (QUANTA 200 instrument (Philips-FEI, Holland)) at 20.0 kV. The sample was immersed in water for one week and freeze-dried before test.

***Fourier-transform infrared spectrometry (FTIR) characterization:*** The chemical structure of the hydrogel sample was measured by Thermo Nicolet-6700 Fourier Infrared spectrometer. The sample was dried in a vacuum oven at 60 ^o^C, followed by grounding into powder with KBr. The test range was 400-4000 cm^-1^, test resolution was 4 cm^-1^, and number of scans was 32 times.

***Water contact angle measurement:*** The DSA100 (KRU̎SS, Germany) was used to measure the water contact angles of the sample, where the water drop size was 5 μL.

***Ultraviolet-visible-near-infrared spectrum (UV-Vis-NIR) measurement:*** The UV-Vis-NIR analysis was conducted by UV 3600 with the range of 200-2500 nm, to evaluate the absorption capacity of full spectrum light source of the hydrogel evaporator.

***Ultraviolet-visible spectrum (UV-Vis) measurement:*** The UV-visible spectrum of the organic dye before and after purification was measured by Lambda 900 in the range of 200-800 nm.

***Water evaporation measurement:*** The hydrogel sample was immersed in water to remove the unreacted monomers and solvent before water evaporation. Each evaporation measurement was performed three times to ensure data accuracy. (1) For pure water evaporation, xenon lamps were used to simulate sunlight (light intensity of 1 kW/m^2^). A cylindrical container with radius of 4.5 cm and height of 2 cm is used hold deionized water, where the top of the container was covered with a polystyrene foam (with a hole in center to hold the hydrogel evaporator). The hydrogel evaporator crossed through the hole and immersed in the deionized water. Under the exposure to xenon lamp, the temperature at the top of the evaporator and the mass change of deionized water was recorded every 10 minutes by an infrared thermal imager and an electronic balance. (2) For salt water evaporation, the deionized water in pure water evaporation was replaced by 5 wt%, 10 wt% and 15 wt% of NaCl solutions. (3) For organic dye evaporation, the deionized water in pure water evaporation was replaced by RhB, MO and MB solution with a concentration of 100 mg/L.

***Statistical analyses***

All the experiments were done in triplicates for repeatability. Results were expressed as means ±SD of three independent experiments.

**2. Supplementary Figures**

**Figure S1.** Storage modulus (G') and loss modulus (G'') of ink versus shear stress at DMAPS/AM mass ratio of 1:3 and 2:2 (w/w).

**Figure S2.** Storage modulus G' and loss modulus G'' under dynamic cyclic strain sweeps at DMAPS/AM mass ratio of 1:3 and 2:2 (w/w).

**Figure S3.** (a) Frontal position-time curves and (b) Fontal velocity and *T_max_* at varying DMAPS/AM mass ratios in glass tube.

**Figure S4.** Swelling behavior of hydrogels with varying DMAPS/AM mass ratios in water.

**Figure S5.** SEM images of hydrogels at varying DMAPS/AM mass ratios

**Figure S6.** Water-transfer capacity of bulk and 3D grid hydrogel.

**Figure S7.** Swelling behavior of hydrogels with varying salinity at DMAPS/AM mass ratio of 3:1 (w/w)

**Figure S8.** Water contact angle of the hydrogel with varying DMAPS/AM mass ratios (water) and salinity (DMAPS/AM = 3:1 (w/w)).


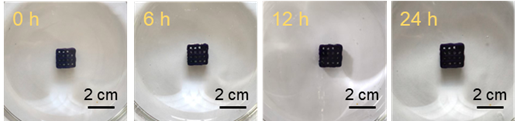


**Figure S9.** Dimensional stability during prolonged immersion in 15 % salt water.


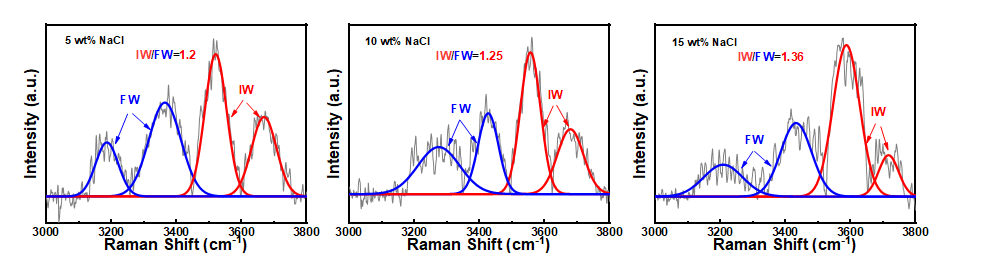


**Figure S10.** Raman spectra of hydrogel at varying salinity (DMAPS/AM mass ratio of 3:1 (w/w)).


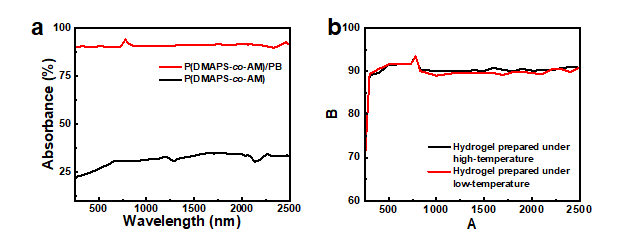


**Figure S11.** (a) UV-Vis-NIR absorption spectra of poly(DMAPS-*co*-AM)/PB and poly(DMAPS-*co*-AM) hydrogel evaporator. (b) UV-Vis-NIR absorption spectra of poly(DMAPS-*co*-AM)/PB prepared under high temperature (FP method) and room temperature (spontaneous polymerization).

**Figure S12.** Surface temperature change of the poly(DMAPS-co-AM)/PB hydrogels with different DMAPS/AM mass ratios of 1:3, 2:2 and 3:1 (w/w).

**Figure S13.** Photographs of the hydrogel sample after 7h evaporation in 15 wt% brine.


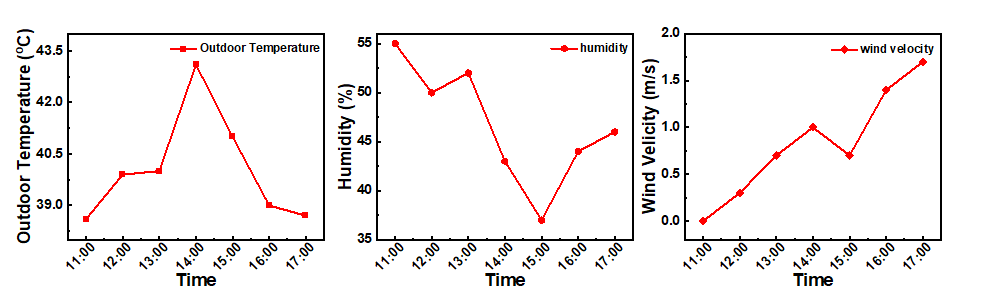


**Figure S14.** Outdoor temperature, humidity, wind speed diagram.

**Figure S15.** UV-Vis absorption spectra of organic dye before and after purification: MB, RhB and MO.
